# Supplementary material for: Systemic brain tumor delivery of synthetic protein nanoparticles for glioblastoma therapy
Source: Nat Commun. 2020 Nov 10;11:5687. doi: 10.1038/s41467-020-19225-7 (PMC7655867; doi:10.1038/s41467-020-19225-7)
Supplement: Supplementary file 1 — Supplementary Information [file 41467_2020_19225_MOESM1_ESM.pdf]

Systemic Brain Tumor Delivery of Synthetic Protein Nanoparticles for Glioblastoma Therapy

Gregory et al.

Supplementary Information:

Supplementary Table 1
Supplementary Methods Tables 2 - 8
Supplementary Figures 1 - 19

Supplementary Tables

Supplementary Table 1. Mouse serum biochemical analysis following intravenous STAT3*i* SPNP + IR treatment
23 DPI (n = 3)

Group	Creatinine (μM)	BUN (mM)	ALT (U L^{-1})	AST (U L^{-1})
Saline	0.21 ± 0.10	26 ± 3.1	111 ± 45.2	328 ± 31.4
STAT3*i*	0.20 ± 0.04	23 ± 5.0	98 ± 9.2	331 ± 40.2
SPNPs	0.23 ± 0.10	27 ± 2.1	102 ± 4.0	342 ± 32.1
STAT3*i* SPNPs	0.17 ± 0.20	23 ± 3.0	97 ± 3.5	332 ± 28.2
STAT3*i* SPNPs + IR	0.20 ± 0.06	28 ± 1.2	99 ± 2.6	320 ± 45.0

Supplementary Methods

Supplementary Table 2. Particle synthesis materials

Material	Source	Catalog number
Human serum albumin	Sigma Aldrich	A3782
O,O′-Bis[2-(N-Succinimidyl-succinylamino)ethyl]polyethylene glycol	Sigma Aldrich	713783
ethylene glycol	Sigma Aldrich	324558
UltraPure DNase/RNase-free distilled water	Thermo Fisher	10977015
Albumin from bovine serum Alexa Fluor 647cConjugate	Thermo Fisher	A24785
Albumin from bovine serum Alexa Fluor 488 conjugate	Thermo Fisher	A13100
Silencer GFP (eGFP) siRNA	Thermo Fisher	AM4626
Silence Cy3-labeled negative control siRNA	Thermo Fisher	AM4621
SignalSilence STAT3 siRNA I mouse	Cell Signaling	6353
Polyethyleneimine	Sigma Aldrich	181978
iRGD peptide	Ruoslahti Lab	N/A

Supplementary Table 3. Capillary immunoassay reagents / antibodies

Material (Antibody / Dilution)	Source	Catalog number
Beta Actin (13E5) rabbit mAb<br>1:50	Cell Signaling	4970
GAPDH (14C10) rabbit mAb<br>1:50	Cell Signaling	2118S
STAT3 (D3Z2G) rabbit mAb<br>1:50	Cell Signaling	12640
Phospho-STAT3 (Tyr705) (D3A7) XP rabbit mAb<br>1:50	Cell Signaling	9145

Supplementary Table 4. Immunofluorescence reagents

Material	Source	Catalog number
Anti-LAMP1 antibody [EPR21026]<br>1:100	abcam	ab208943
Goat anti rabbit IgG HL Alexa Fluor 555<br>1:200	abcam	ab150078
Prolong diamond antifade mountant with DAPI	Thermo Fisher	P36966

Supplementary Table 5. Flowcytometry antibodies

Antibodies / Dilution	Source	Catalog number
V450 rat anti mouse CD45<br>1:200	BD Bioscience	560501
PE Rat anti mouse F4/80<br>1:200	Biolegend	123110
Alexa Fluor 700 rat anti mouse CD206<br>1:200	Biolegend	9661
PE Armenian hamster anti mouse CD11C<br>1:200	Biolegend	117308
PercpCy5.5 rat anti mouse B220<br>1:200	Biolegend	103236
FITC rat anti mouse CD8<br>1:200	Biolegend	100706
PercpCy5.5 Armenian hamster anti mouse CD3<br>1:200	Biolegend	100328
PB rat anti mouse Granzyme B<br>1:200	Biolegend	515407
PE rat anti mouse IFNγ<br>1:200	Biolegend	505808
PE-Tetramer<br>1:100	MBL International	TB-5001-1
Efluor 780-Live/Dead<br>1:200	Affymetrics	65-0865-14

Supplementary Table 6. Immunohistochemistry primary antibodies

Antibodies / Dilution	Source	Catalog number
Rat anti mouse MBP<br>1:300	Millipore	MAB386
Rabbit anti mouse CD8<br>1: 2000	Cedarlane	361003
Rat anti mouse F4/80<br>1:500	BioRad	MCA497RT

Supplementary Table 7. Immunohistochemistry secondary antibodies

Antibodies / Dilution	Source	Catalog number
Goat polyclonal anti rabbit biotin-conjugated<br>1:1000	Dako	E0432
Goat polyclonal anti rat Alexa Fluor 594<br>1:1000	Thermo Fisher	A-11007
Goat polyclonal anti rabbit biotin-conjugated<br>1:1000	Thermo Fisher	31830

Supplementary Table 8. Reagents for cell culture

Reagent	Source	Catalog number
Dulbecco's modified eagle medium (DMEM)	Thermo Fisher	12430054
Heat-inactivated fetal bovine serum (FBS)	Atlanta Biologicals	S11150
Penstrep (10,000U mL^{-1})	Thermo Fisher	15140122
G418	Thermo Fisher	10131035
Trypsin-EDTA (0.05%), phenol red	Thermo Fisher	25300054
DPBS, no calcium and no magnesium	Thermo Fisher	14190144

Supplementary Figures

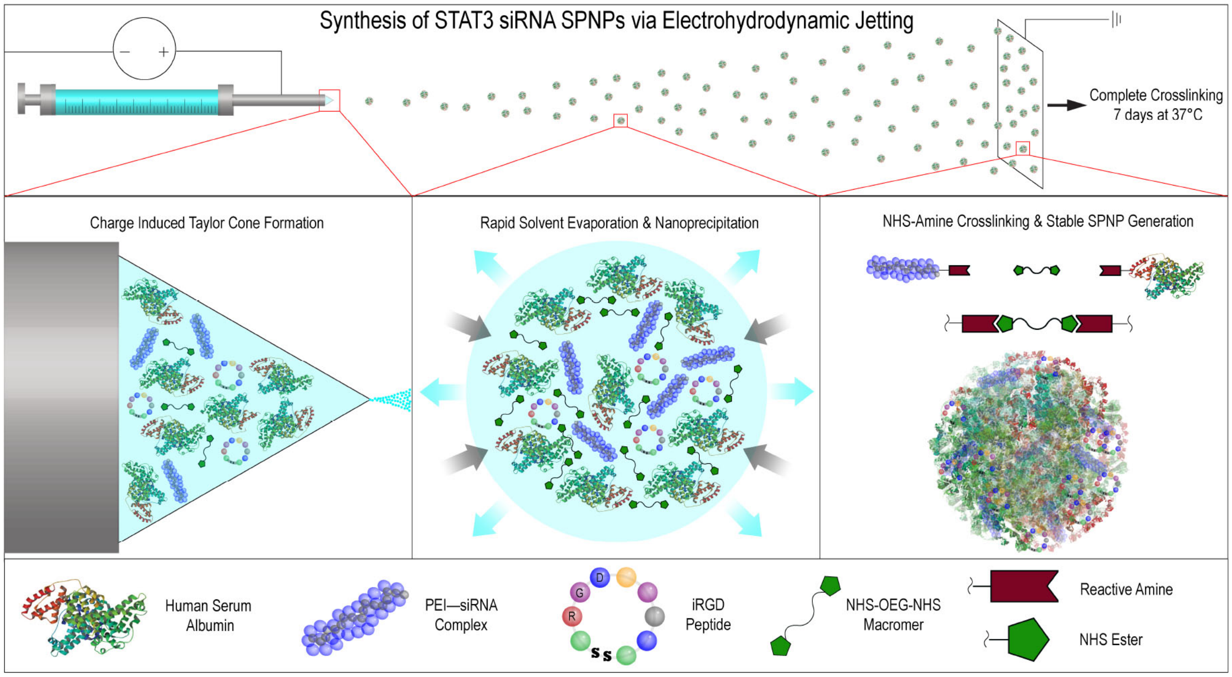

Supplementary Figure 1. Synthesis of STAT3*i* SPNPs. Electrohydrodynamic (EHD) jetting uses a dilute solution of all components to be incorporated into the ultimate protein nanoparticle (here: HSA, OEG macromer, STA3*i*-PEI complex, and iRGD in an aqueous system). Using controlled flow through a single capillary, the application of an electric field distorts the droplet to form a stable Taylor Cone from which a jet of charged droplets emanates. Once atomized, rapid evaporation of the solvent induces nearly instantaneous nanoprecipitation of all non-volatile components to form solid protein nanoparticles. The bifunctional OEG macromer covalently links the protein and PEI units through reactive amine groups, resulting in a continuous network. The STAT3*i* is complexed to the PEI through electrostatic interactions, while the iRGD is encapsulated.

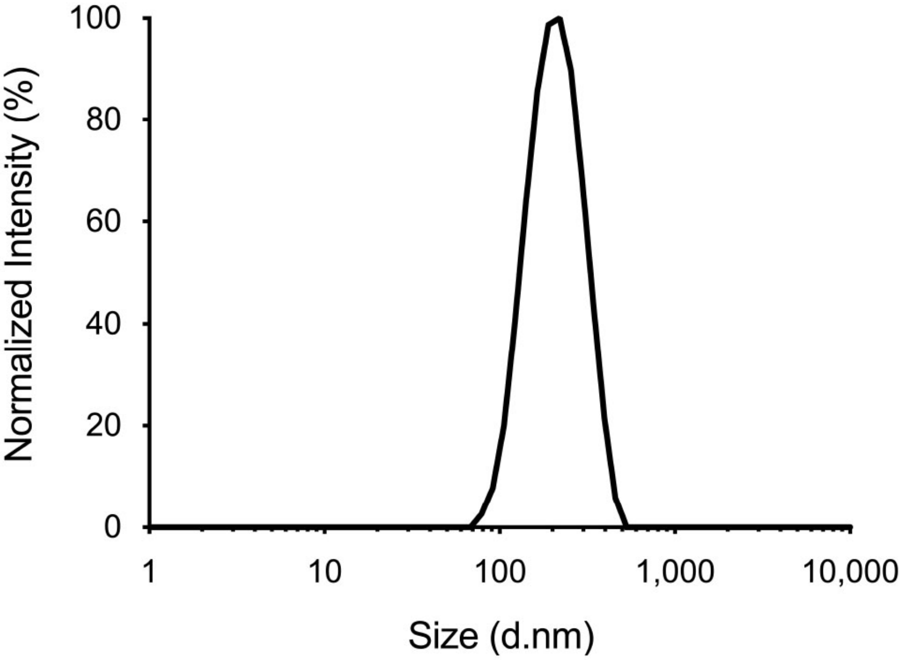

Supplementary Figure 2. SPNPs are stable at physiological conditions. Characterization of SPNPs size was measured by dynamic light scattering (DLS) following the collection process and storage in PBS (pH 7.4) at 4°C. Particles were observed to swell with an increase in average size compared to their dry, crosslinked state. Average diameter = 220 nm. No change was observed in average particle diameter over a period of 1 month at the above conditions.

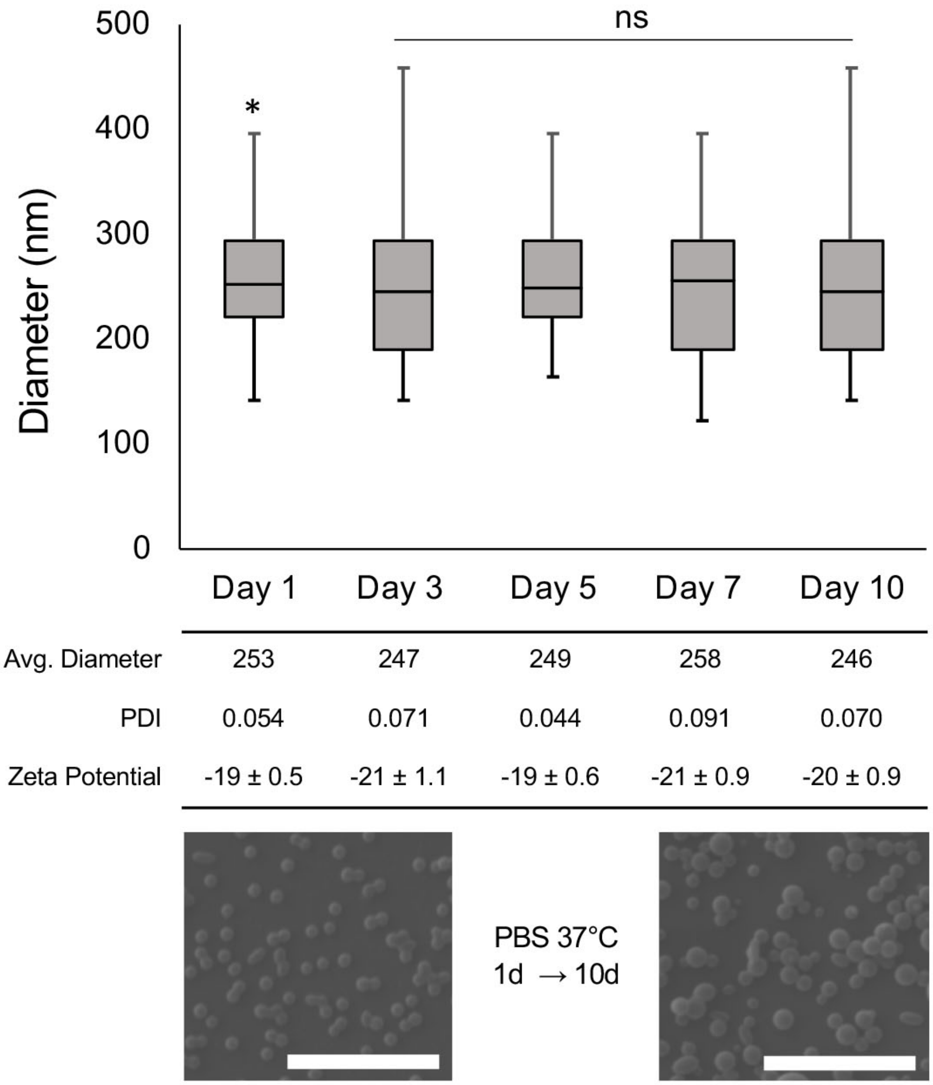

	Day 1	Day 3	Day 5	Day 7	Day 10
Avg. Diameter	253	247	249	258	246
PDI	0.054	0.071	0.044	0.091	0.070
Zeta Potential	-19 ± 0.5	-21 ± 1.1	-19 ± 0.6	-21 ± 0.9	-20 ± 0.9

Supplementary Figure 3. SPNPs remain stable in solution under relevant physiological conditions. (Top) After a single day in PBS at 37°C, SPNPs show no significant change in particle size as measured by dynamic light scattering (DLS). Particles appear to both remain intact and do not aggregate under these conditions. Similarly, the polydispersity index (PDI) and zeta potential values remain consistent over the ten day incubation period. Hydrodynamic diameter data from n = 5 technical replicates was collected and averaged provided the data met minimum quality standards including a correlation function with a single inflection point. Box and whisker plot data are presented as mean values, first and third quartiles (box) and minimum/maximum values (whiskers), Statistical significance determined with one-way ANOVA and Tukey's multiple comparison test, * $p = 0.044$. (Bottom) No change in particle shape or morphology is observed following the incubation at physiological conditions as imaged by scanning electron microscopy (SEM). Scale bars = 2 μm.

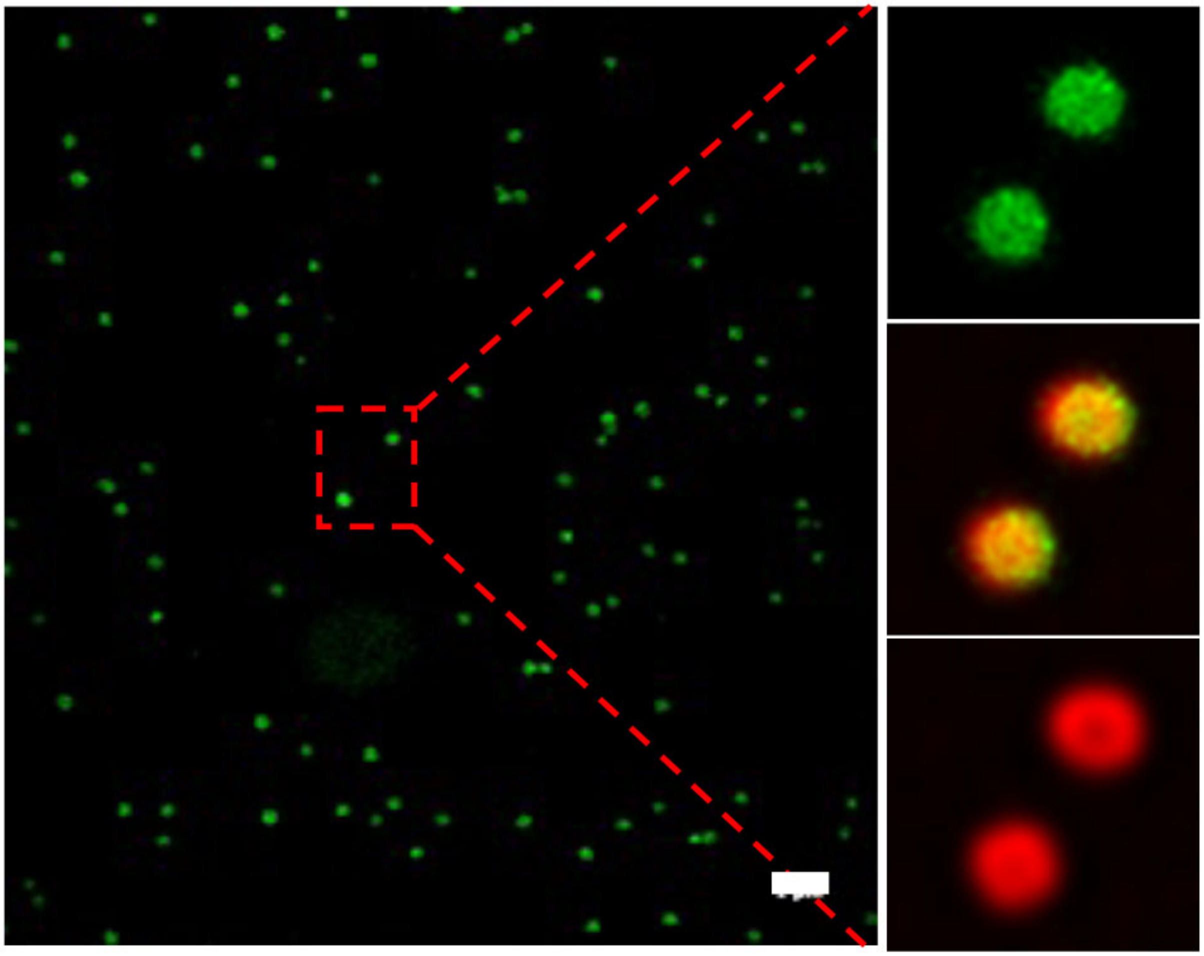

Supplementary Figure 4. SPNPs loaded with Cyanine3-siRNA exhibit controlled siRNA release. Alexa Fluor 488 (green) labeled SPNPs were loaded with a fluorescently (Cy3, red) labeled, scrambled siRNA. Several images were collected across a single prepared sample using super-resolution, Stimulation Emission Depletion (STED) microscopy. Colocalization of the two signals was used to confirmed the encapsulation of siRNA within the particles. Scale bar = 1 μm.

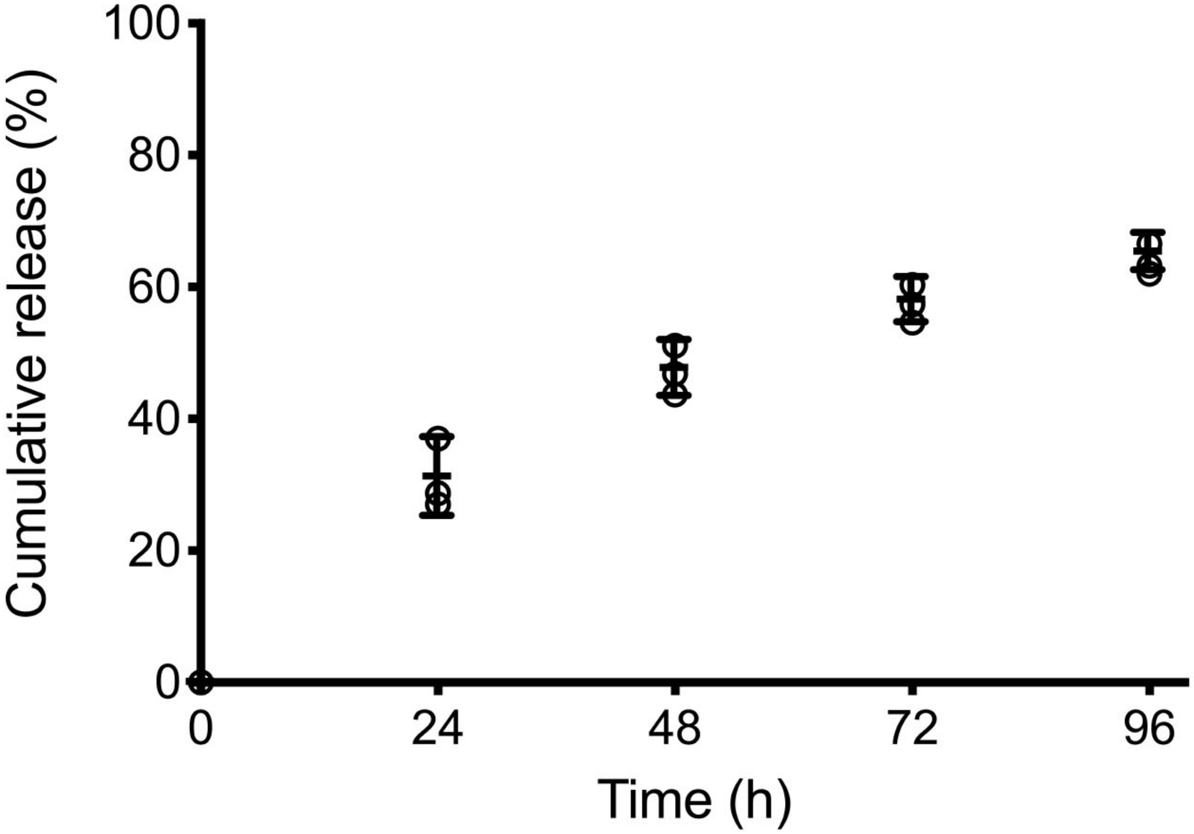

Supplementary Figure 5. Controlled siRNA release from SPNPs. Loading and subsequent release of Cy3-labeled scrambled siRNA demonstrates a controlled and extended release of incorporated NP cargo. While 60% of the initially encapsulated siRNA is released in the first 96 hours at pH 7.4 and 37°C, continued release is observed for up to 21 days. Complete release confirms a loading efficiency of 96%. Data are presented as mean values ± s.d. (n = 3 biological replicates).

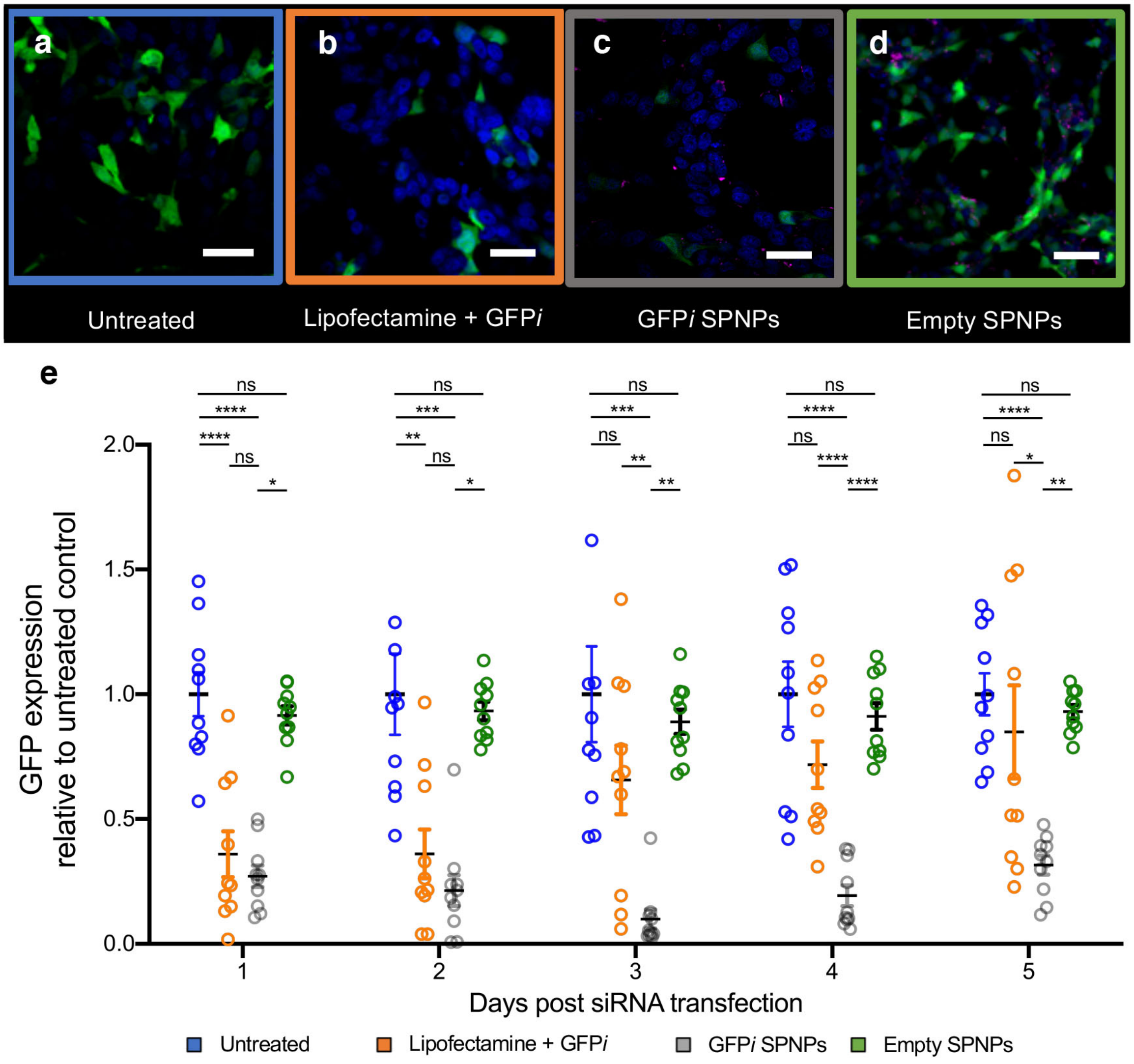

Supplementary Figure 6. *in vitro* GFP siRNA-loaded SPNPs reduce target protein expression. (a)-(d) Representative Confocal Scanning Laser Microscopy (CSLM) images of GL26-Cit cells incubated with NPs at 48 h time point. (a) Control group receiving no treatment. (b) Positive control group transfected with GFP siRNA (GFP*i*) using Lipofectamine 2000. (c) Cells treated with GFP*i* loaded nanoparticles at a concentration of 25 µg NPs per mL. (d) Cells treated with empty albumin nanoparticles. **(e)** GFP expression plotted relative to untreated control group over a period of 5 days. A significant and prolonged suppression of target protein is observed in cells that received the siRNA-loaded nanoparticles. A similar knockdown was observed in cells transfected with free siRNA using Lipofectamine at early time points, but a rapid recovery was observed after 48 h. Data are presented as mean values ± SEM relative to untreated control (n = 10 independent composite images; two-tailed, unpaired t-tests; **** $p < 0.0001$, *** $p < 0.001$, ** $p < 0.01$, * $p(1) = 0.028$, * $p(2) = 0.007$, * $p(5) = 0.012$). Scale bars = 50 µm.

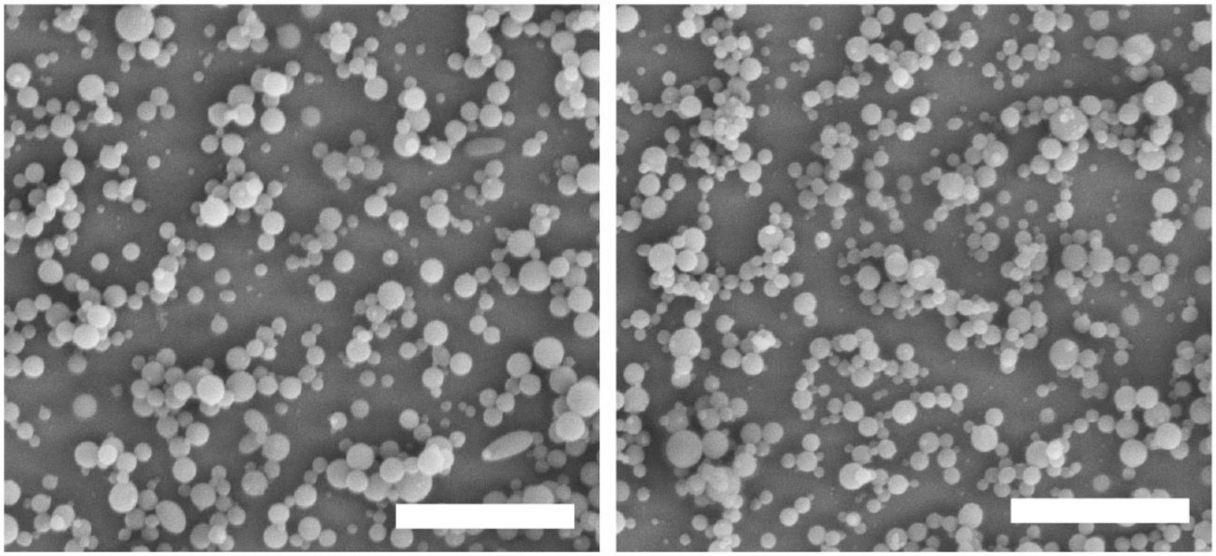

Supplementary Figure 7. siRNA-loaded SPNPs exhibit similar size and morphology to control SPNPs. The addition of the siRNA/PEI complex to the jetting solution to create siRNA-loaded SPNPs (left) results in no significant change in particle size, shape or surface morphology when compared to control (empty) SPNPs (right). When suspended in PBS, no significant difference in zeta potential (siRNA-loaded SPNPs $\zeta = -20 \pm 1.2$, Control SPNPs $\zeta = -19 \pm 2.9$) was observed. Imaging and characterization was performed following the synthesis of particles for all in vitro and in vivo experiments. Across ten independently synthesized batches, similar results were observed when comparing SPNPs with and without siRNA/PEI. Scale bars = 2 μm.

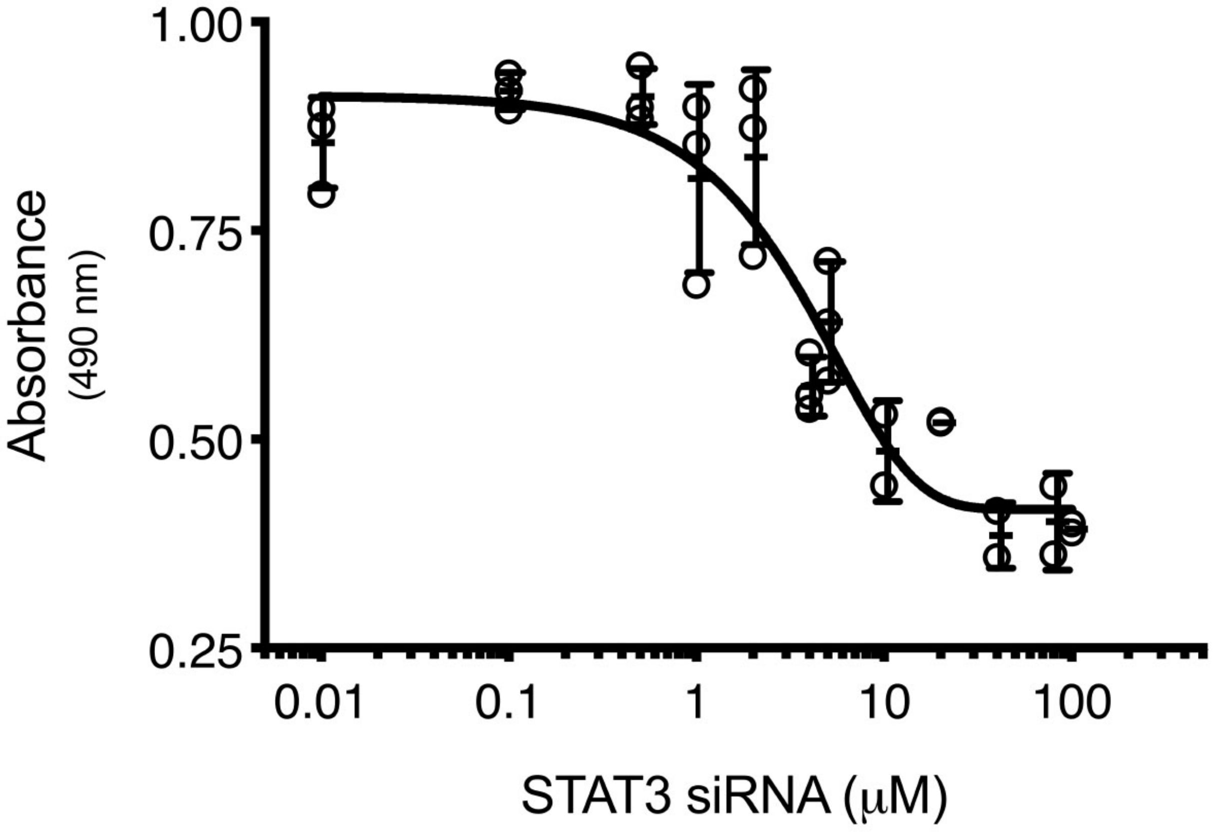

Supplementary Figure 8. STAT3 siRNA activity is non-toxic towards glioma cells. Evaluating toxicity associated with free STAT3*i*, no toxicity was observed at relevant therapeutic concentrations. Concentrations delivered via SPNPs showed a significant silencing ability (6.5 x 10^{-4} μM), 6,000x less than the IC50 (3.85 μM) for soluble siRNA delivered via traditional transfection. Data are presented as mean values ± s.d. (n = 3 biological replicates).

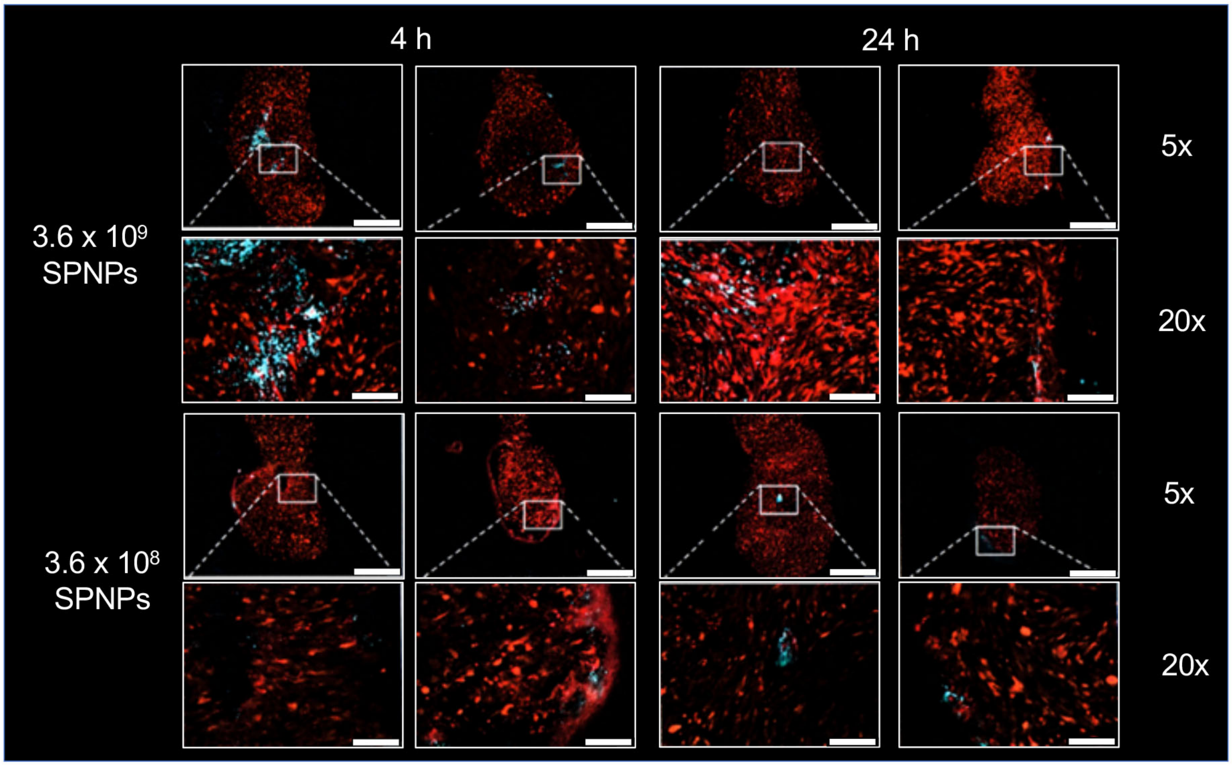

Supplementary Figure 9. SPNPs distribution in the tumor mass following intracranial injection. Following the implantation of m-Tomato expressing GL26 tumors (red), C57BL/6 mice received 3 µL intracranial injections of either 3.6 x 10^8 or 3.6 x 10^9 SPNPs (cyan) per mouse. Images suggest that the particles actively and rapidly distribute throughout the tumor mass. Scale bars = 150 µm (5x), 600 µm (20x).

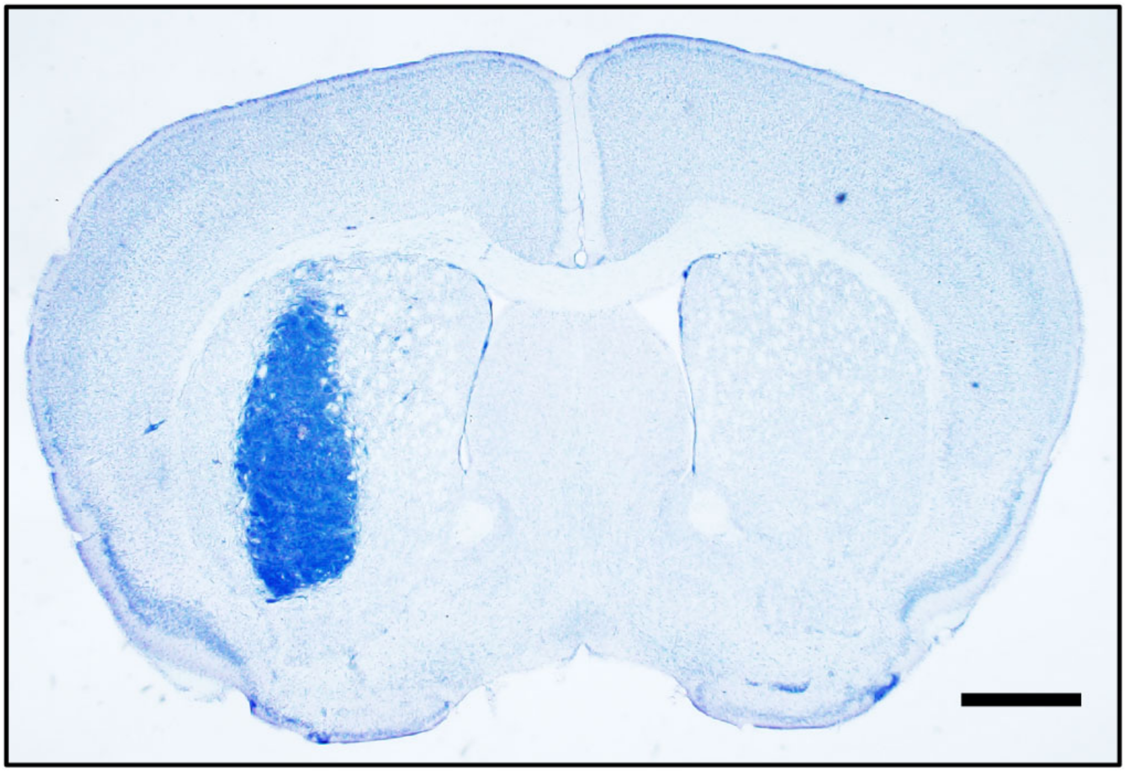

Supplementary Figure 10. Volume of GL26 GBM at 7 DPI. C57BL/6 mice were implanted with 20,000 GL26 cells orthotopically and brains were processed for Nissl staining at 7 DPI. Staining and imaging was conducted as a single independent experiment. Scale bar = 1 mm. Tumor volume = 9.61 mm^3. Tumor area = 107 pixel units.

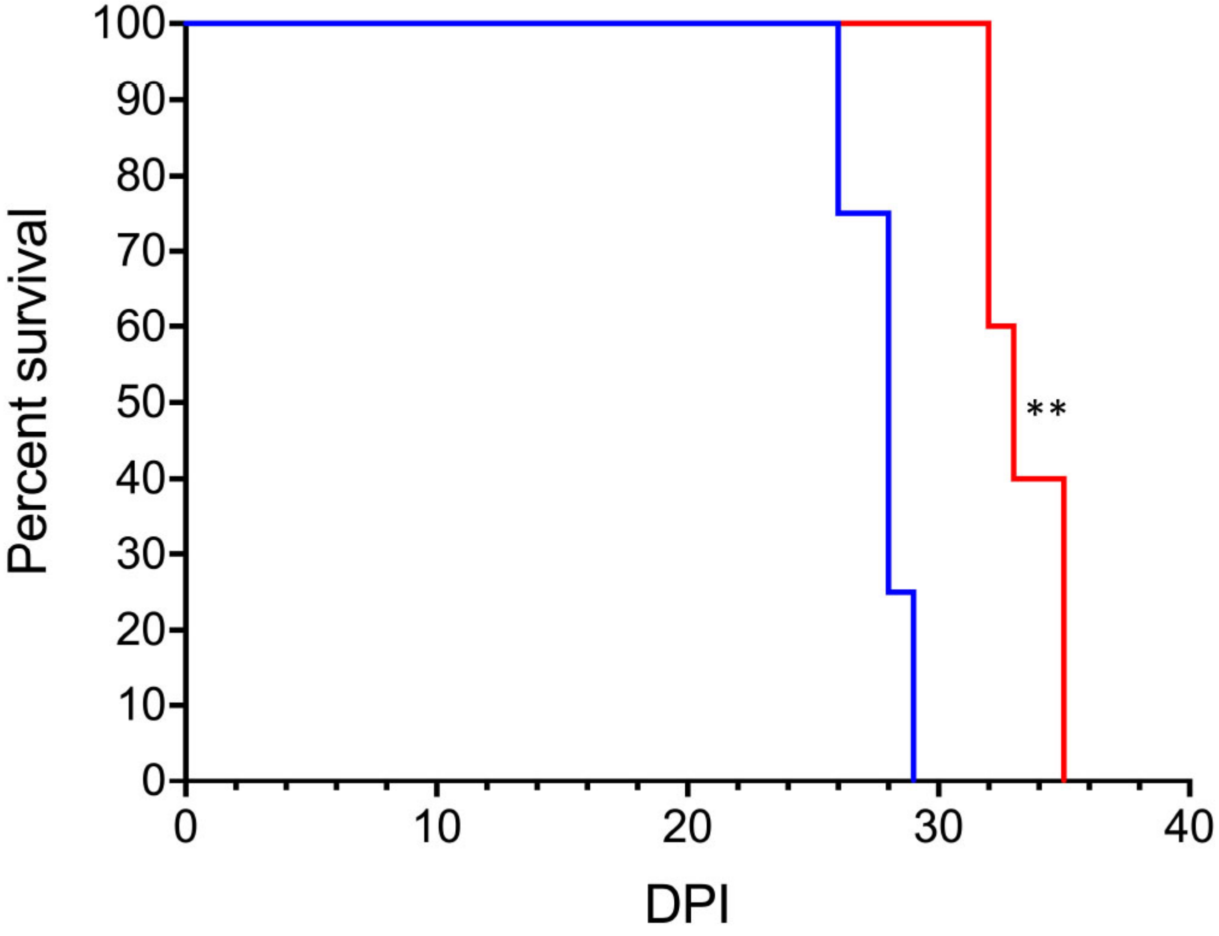

Supplementary Figure 11. Kaplan-Meier survival curve for single dose of STAT3*i* SPNPs. C57BL6 mice were implanted with GL26 cells. A single dose of 2.0 x 10^{11} STAT3*i* SPNPs were delivered via tail vein injection five days post tumor implantation. Mice treated with siRNA-loaded particles (red, n = 5) had a median survival of 33 days, 5 days longer than mice in the saline treated control group (blue, n = 4). Data were analyzed using the log-rank (Mantel-Cox) test. ** p = 0.0029, MS = median survival.

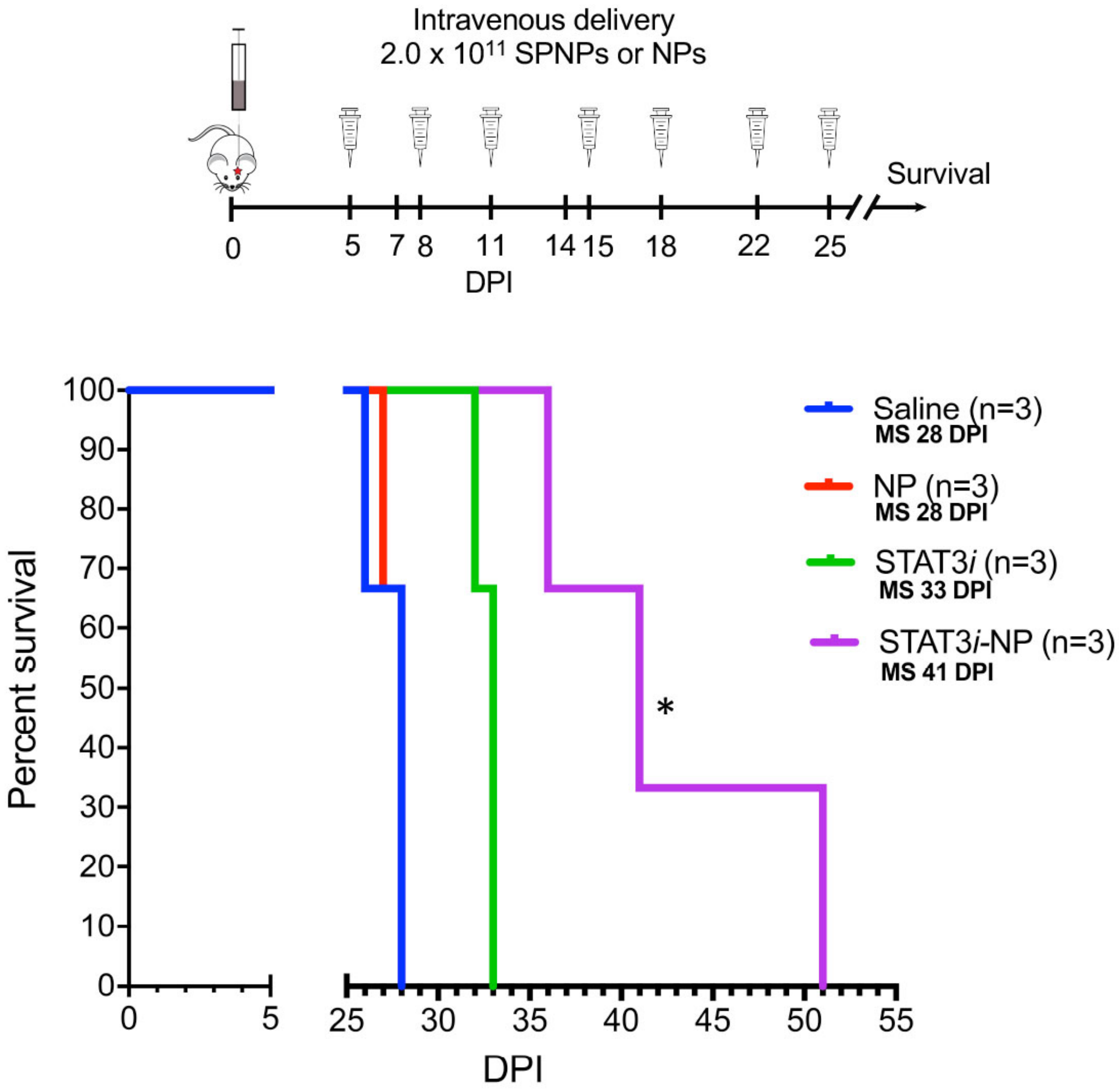

Supplementary Figure 12. Three-week STAT3*i* SPNP treatment regimen extends median survival. C57BL/6 mice were implanted with GL26 cells, at 5 DPI mice were treated with 2.0 x 10^{11} STAT3*i* SPNPs. STAT3*i* SPNPs delivered via tail vein (IV) injection. STAT3*i* SPNPs elicited a 46% increase in median survival compared to saline treated control mice (MS = 41 vs. 28). Soluble IV administered STAT3*i* showed a moderate therapeutic effect (MS = 33 vs. 28) while mice treated with vehicle SPNPs saw no effect. Data were analyzed using the log-rank (Mantel-Cox) test. * $p = 0.0052$, MS = median survival.

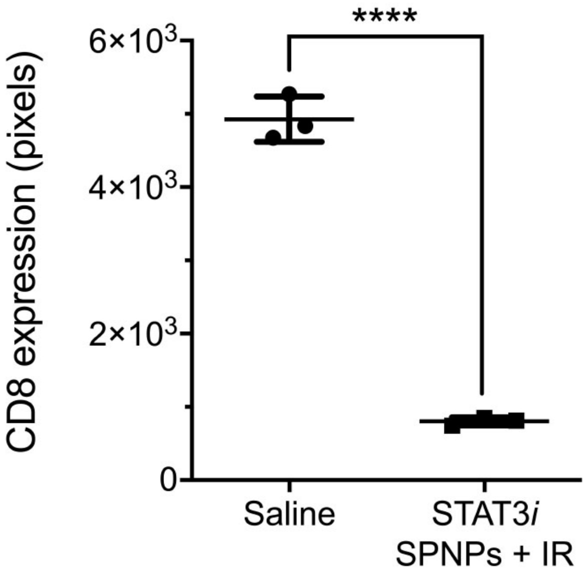

Supplementary Figure 13. Quantification of CD8 Expression in TME. Immunofluorescence staining of tumors in either saline or STAT3*i* SPNP + IR treatment groups was quantified using otsu threshold by ImageJ. Data represent total number of positive cells for CD8 in saline (28 DPI) versus. STAT3*i* SPNPs + IR (90 DPI) long-term survivor. Data are presented as mean ± s.d. (n = 3 biological replicates; two-tailed unpaired t-test; **** $p < 0.0001$).

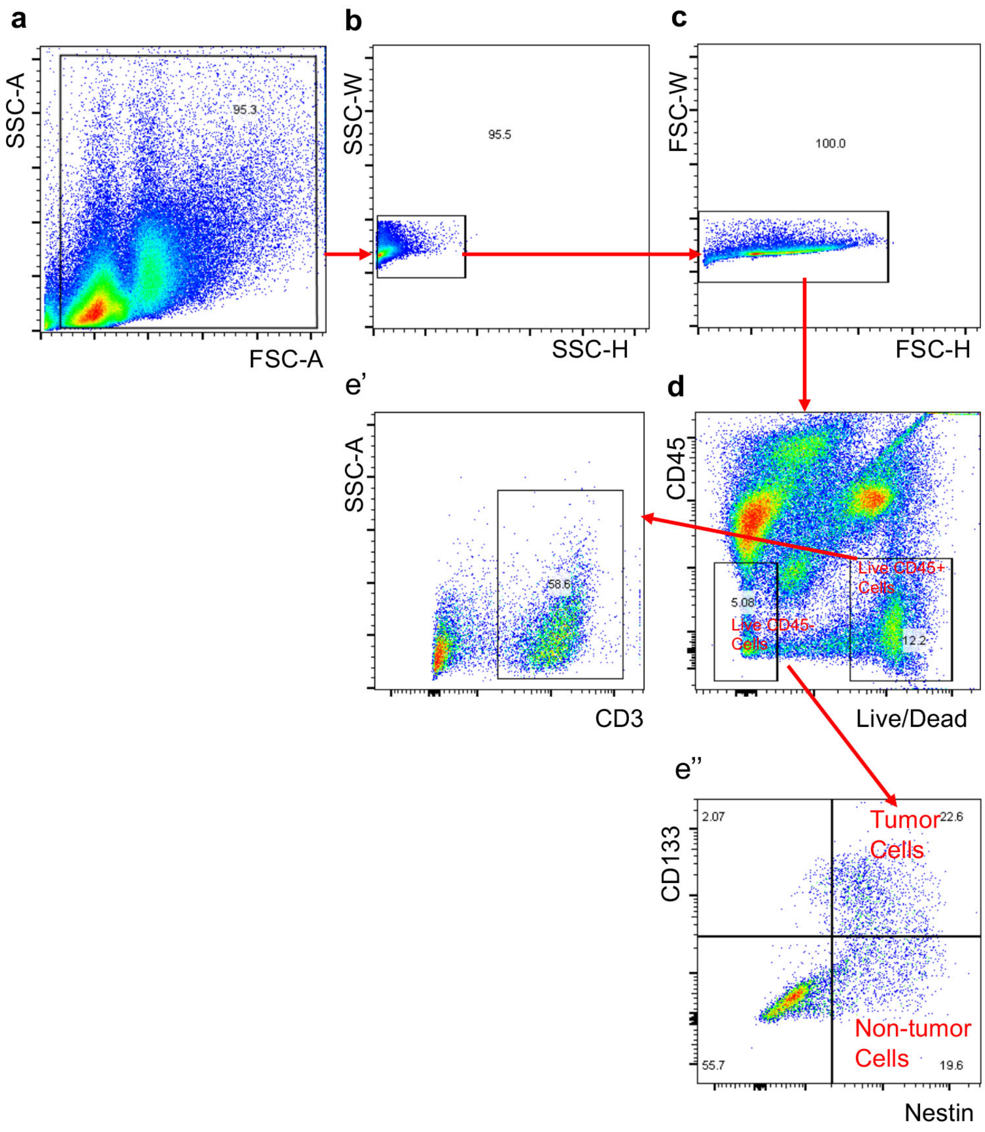

Supplementary Figure 14. Sequential gating strategy for flow cytometry analysis of immunolabeled cells. (a) Immunolabeled cells were gated to exclude cellular debris. **(b)-(c)** Doublet discrimination gating was performed to filter out cellular aggregates prior to analysis. **(d)** $CD45^+$/Live cells were gated to identify GBM infiltrating M1 ($CD45^+$/$F480^+$/$CD206^-$), and M2 ($CD45^+$/$F480^+$/$CD206^+$) macrophages shown in Fig. 5a as well as cDCs ($CD45^+$/$CD11c^+$/$B220^-$) shown in Fig. 5b. $CD45^-$/Live cells were gated to identify tumor cells. **(e')** Identification of live $CD3^+$ cells from the $CD45^+$/Live parent gate in (d) to determine the amount of GBM specific T cells ($CD45^+$/$CD3^+$/$CD8^+$/ H2Kb-OVA tetramer+), and cytotoxic T cells ($CD45^+$/$CD3^+$/$CD8^+$/ IFN-y or $CD45^+$/$CD3^+$/$CD8^+$/ granzyme B) in the TME shown in Fig. 3f. **(e'')** Identification of $CD133^+$/$nestin^+$ GBM cells from the $CD45^-$/live parent gate in (d) for avβ3 and avβ5 integrin expression analysis shown in Fig. 1f.

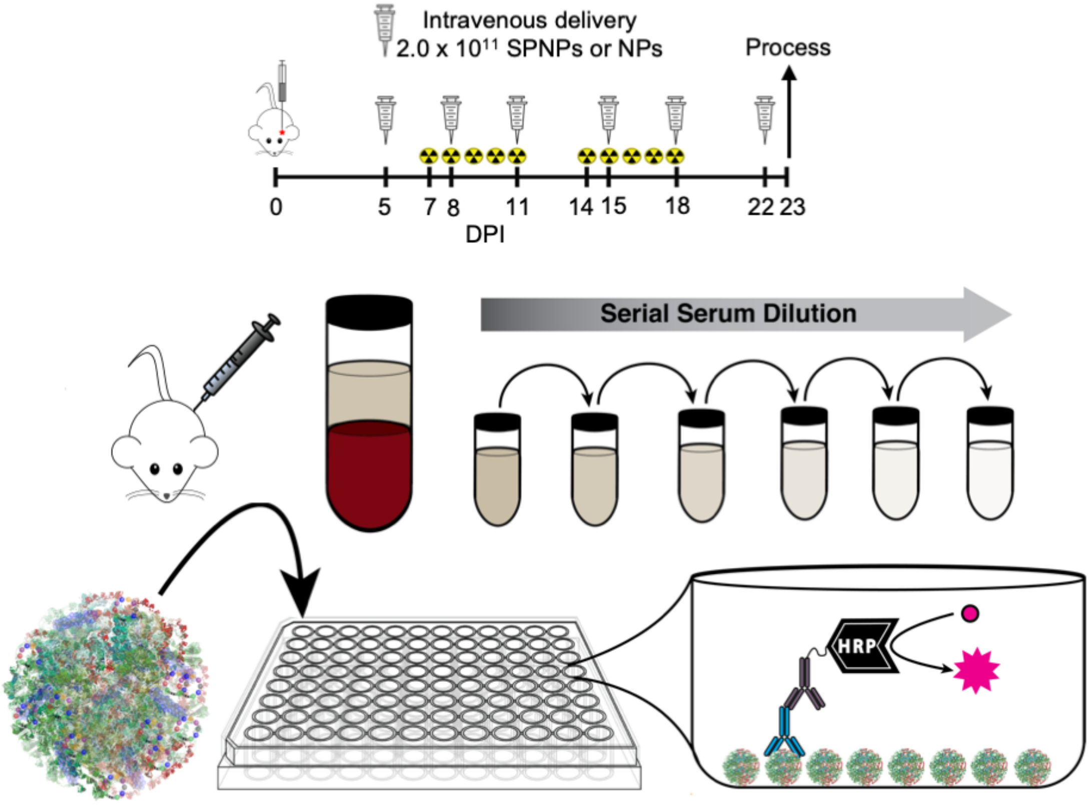

Supplementary Figure 15. Evaluation of circulating antibodies against STAT3*i* SPNPs. Following complete STAT3*i* SPNPs + IR treatment, a modified ELISA protocol was used to detect the presence of circulating antibodies against SPNPs in serum of GL26 tumor bearing mice.

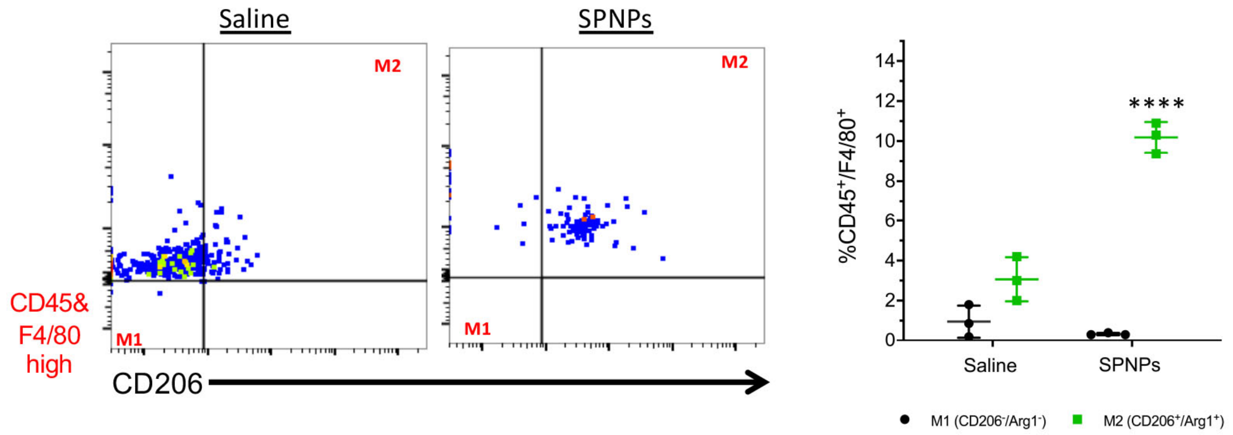

Supplementary Figure 16. SPNPs treatment induces a shift in the TME macrophage balance. Treatment of GL26 tumor-bearing mice with empty SPNPs produced a shift in macrophage populations within the tumor microenvironment. An increase in the M2 macrophages relative to saline treated control animals was observed. Data are presented as mean values ± s.d. ($n = 3$ biological replicates; two-way ANOVA; **** $p < 0.0001$)

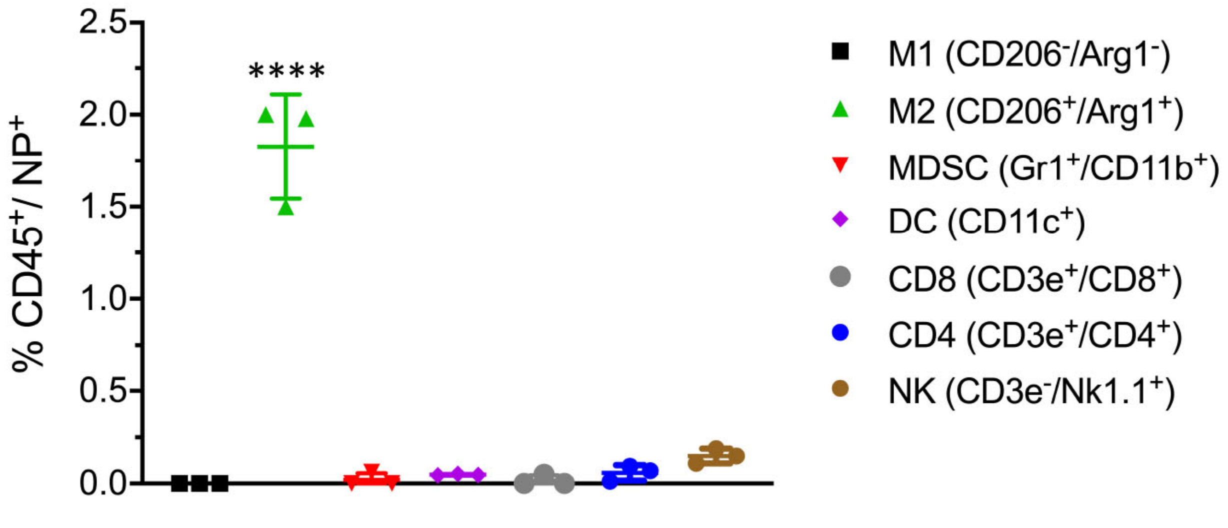

Supplementary Figure 17. Among TME immune cells, only M2 macrophages showed significant uptake of SPNPs. Flow cytometry analysis of immune cells collected from the tumor microenvironment of SPNPs treated mice show significant nanoparticle uptake by M2 macrophages and minimal uptake by all other immune cell types. Data are presented as mean values ± s.d. (n = 3 biological replicates; two-way ANOVA; **** $p < 0.0001$).

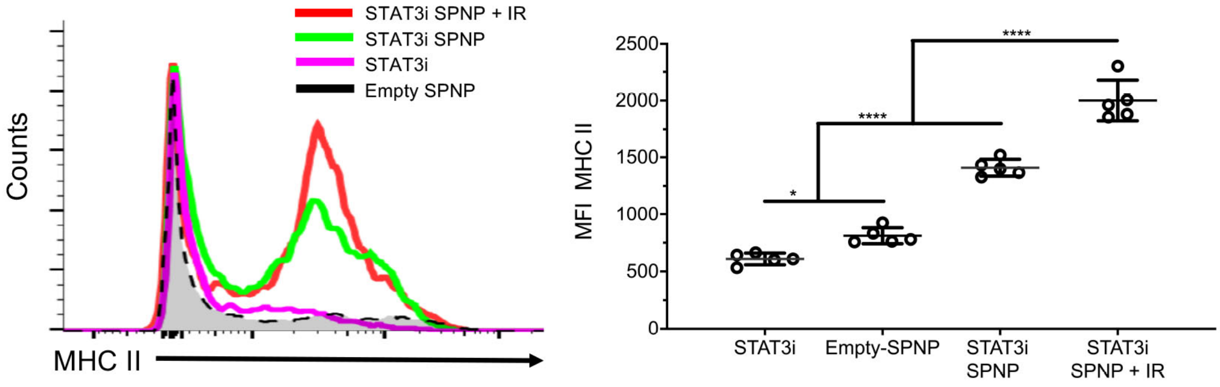

Supplementary Figure 18: Dendritic cell MHC II expression. Activation status of DCs in the draining lymph node of GBM bearing mice treated with STAT3*i*, empty SPNP, STAT3*i* SPNP, and STAT3*i* SPNP + IR was assessed one day post the last day of treatment (23 DPI). Representative histograms display MHC II expression level on the DCs (purple = STAT3*i*, black = empty SPNP, red = STAT3*i* SPNP + IR, green = STAT3*i* SPNP). Data is presented as mean values ± s.d. (n = 5 biological replicates; one way ANOVA; **** $p < 0.0001$, * $p = 0.036$).

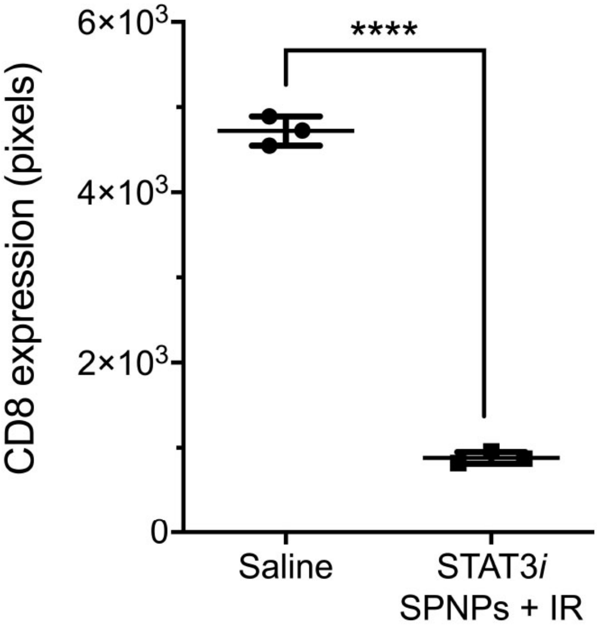

Supplementary Figure 19. Quantification of CD8 expression in rechallenged long-term survivor TME. Immunofluorescence staining of tumors in each treatment group was quantified using otsu threshold by ImageJ. Data represent total number of positive cells for CD8 in saline (28 DPI) versus STAT3*i* SPNPs + IR (90 DPI post rechallenged; 180 DPI post initial tumor implantation) rechallenged long-term survivor. Data are presented as mean values ± s.d. (n = 3 biological replicates; two-tailed, unpaired t-test; **** $p < 0.0001$).
