# Supplementary material for: Systemic brain tumor delivery of synthetic protein nanoparticles for glioblastoma therapy
Source: Nat Commun. 2020 Nov 10;11:5687. doi: 10.1038/s41467-020-19225-7 (PMC7655867; doi:10.1038/s41467-020-19225-7)
Supplement: Supplementary file 3 — Reporting Summary [file 41467_2020_19225_MOESM3_ESM.pdf]

nature research

Corresponding author(s): Joerg Lahann
Maria G. Castro

Last updated by author(s): Aug 13, 2020

Reporting Summary

Nature Research wishes to improve the reproducibility of the work that we publish. This form provides structure for consistency and transparency in reporting. For further information on Nature Research policies, see our Editorial Policies and the Editorial Policy Checklist.

Statistics

For all statistical analyses, confirm that the following items are present in the figure legend, table legend, main text, or Methods section.

n/a	Confirmed	
☐	☒	The exact sample size (n) for each experimental group/condition, given as a discrete number and unit of measurement
☐	☒	A statement on whether measurements were taken from distinct samples or whether the same sample was measured repeatedly
☐	☒	The statistical test(s) used AND whether they are one- or two-sided<br>*Only common tests should be described solely by name; describe more complex techniques in the Methods section.*
☒	☐	A description of all covariates tested
☐	☒	A description of any assumptions or corrections, such as tests of normality and adjustment for multiple comparisons
☐	☒	A full description of the statistical parameters including central tendency (e.g. means) or other basic estimates (e.g. regression coefficient) AND variation (e.g. standard deviation) or associated estimates of uncertainty (e.g. confidence intervals)
☐	☒	For null hypothesis testing, the test statistic (e.g. F, t, r) with confidence intervals, effect sizes, degrees of freedom and P value noted<br>*Give P values as exact values whenever suitable.*
☒	☐	For Bayesian analysis, information on the choice of priors and Markov chain Monte Carlo settings
☒	☐	For hierarchical and complex designs, identification of the appropriate level for tests and full reporting of outcomes
☒	☐	Estimates of effect sizes (e.g. Cohen's d, Pearson's r), indicating how they were calculated

Our web collection on statistics for biologists contains articles on many of the points above.

Software and code

Policy information about availability of computer code

Data collection	Nikon NIS-Elements AR (Version 5.11.00)<br>Leica LAS X (Version 3.7)<br>Malvern Zetasizer Nano (Version 7.13)<br>Malvern NTA (Version 3.30)<br>Flow Jo (Version 10)<br>Compass for SW (Version 4.1)
Data analysis	ImageJ (Version 2.0.0-rc-69/1.52p)<br>Compass for SW (Version 4.1)<br>GraphPad Prism (Version 7)<br>Flow Jo (Version 10)<br>Cell Profiler (Version 2.2.0)

For manuscripts utilizing custom algorithms or software that are central to the research but not yet described in published literature, software must be made available to editors and reviewers. We strongly encourage code deposition in a community repository (e.g. GitHub). See the Nature Research guidelines for submitting code & software for further information.

Data

Policy information about availability of data

All manuscripts must include a data availability statement. This statement should provide the following information, where applicable:

- Accession codes, unique identifiers, or web links for publicly available datasets
- A list of figures that have associated raw data
- A description of any restrictions on data availability

The source data underlying all graphical Figs. 1b-c, e-g; 2e-f; 3b-c, g; 4b-i, k-l; 5c, e; and Supplementary Figures 2-3, 5-6, 8, 11-13 and 16-19 are provided as a Source Data file with this paper. All other data supporting the findings of this study are available within the article, its Supplementary Information files, and from the corresponding author upon reasonable request. A reporting summary for this article is available as a Supplementary Information file.

Field-specific reporting

Please select the one below that is the best fit for your research. If you are not sure, read the appropriate sections before making your selection.

☒ Life sciences ☐ Behavioural & social sciences ☐ Ecological, evolutionary & environmental sciences

For a reference copy of the document with all sections, see nature.com/documents/nr-reporting-summary-flat.pdf

Life sciences study design

All studies must disclose on these points even when the disclosure is negative.

Sample size	Following standards of the field, including power analysis, sample sizes were estimated which were capable of yielding statistically significant differences. For in vivo studies, at least n=3 mice were utilized. To verify the data obtained, and estimate the median survival at least three biological replicates were chosen on the basis of previously published studies (PMID: 28129117 and PMID: 30760578, PMID: 27542769). In cases where sample size was less than three due to experimental limitations, statistical differences were not presented.
Data exclusions	No data were excluded from the analyses.
Replication	All in vivo studies were performed a minimum of 2 times and the data were reproducible between each study. Preliminary in vitro studies were completed as single independent experiments.
Randomization	In vitro cell experiments were seeded identically at the onset of the experiments and randomized into the various treatment groups prior to the beginning of treatment protocols. For in vivo studies, once mice were implanted with tumor cells they were randomized into experimental groups of 3-8 mice prior to treatment administration.
Blinding	The investigators were not blinded to group allocations during data collection and analysis of in vitro and in vivo experiments involving various treatments. In order to administer the therapeutic to cells and mice, it was imperative to know their assigned treatment group. In the case of image analysis based experiments (particle uptake and lysosome colocalization) experimental groups were blinded during the imaging portion of the experiment.

Reporting for specific materials, systems and methods

We require information from authors about some types of materials, experimental systems and methods used in many studies. Here, indicate whether each material, system or method listed is relevant to your study. If you are not sure if a list item applies to your research, read the appropriate section before selecting a response.

Materials & experimental systems

n/a	Involved in the study
☐	☒ Antibodies
☐	☒ Eukaryotic cell lines
☒	☐ Palaeontology and archaeology
☐	☒ Animals and other organisms
☒	☐ Human research participants
☒	☐ Clinical data
☒	☐ Dual use research of concern

Methods

n/a	Involved in the study
☒	☐ ChIP-seq
☐	☒ Flow cytometry
☒	☐ MRI-based neuroimaging

Antibodies

Antibodies used	Flowcytometry Antibodies<br><br>V450 rat anti mouse CD45 (clone:30-F11) 1:200 BD Bioscience 560501

PE Rat anti mouse F4/80 (clone: BM8), 1:200 Biolegend 123110
Alexa Fluor 700 Rat anti mouse CD206 (clone:C06862), 1:200 Biolegend 141733
PE armenian hamster anti mouse CD11c (clone:N418), 1:200 Biolegend 117308
PercpCy5.5 rat anti mouse B220 (clone:RA36B2), 1:200 Biolegend 103236
FITC rat anti mouse CD8 (clone: KT15), 1:1:00 Thermofisher MA5-16759
PercpCy5.5 armenian hamster anti mouse CD3 (clone: 145-2C11), 1:200 Biolegend 100328
PB rat anti mouse Granzyme B (clone GB11), 1:200 Biolegend 515407
PE rat anti mouse IFN# (clone XMG1.2, 1:200 Biolegend 505808
PE-Tetramer, 1:100 MBL International TB-5001-1
Efluor 780-Live/Dead, 1:200 Affymetrics 65-0865-14

Immunohistochemistry Primary Antibodies

Rat anti mouse MBP, 1:300 Millipore MAB386
Rabbit anti mouse CD8, 1: 2000 Cedarlane 361003
Rat anti mouse F4/80, 1:500 BioRad MCA497RT

Immunohistochemistry Secondary Antibodies

Goat polyclonal anti-rabbit biotin-conjugated, 1:1000 Dako E0432
Goat polyclonal anti-rat Alexa Fluor 594, 1:1000 Thermo Fisher A-11007
Goat polyclonal anti-rabbit biotin-conjugated, 1:1000 Thermo Fisher 31830

Capillary Immunoassay Antibodies

Beta Actin (13E5) Rabbit mAb 1:50 Cell Signaling 4970
GAPDH (14C10) Rabbit mAb 1:50 Cell Signaling 2118S
STAT3 (D3Z2G) Rabbit mAb 1:50 Cell Signaling 12640
Phospho-STAT3 (Tyr705) (D3A7) XP Rabbit mAb 1:50 Cell Signaling 9145

Immunofluorescence Antibodies

Anti-LAMP1 Antibody [EPR21026], 1:100 abcam ab208943
Goat Anti Rabbit IgG HL Alexa Fluor 555, 1:200 abcam ab150078

ELISA Antibodies

Mouse Anti Human serum albumin, 1:1000 abcam ab10241
Rabbit Anti Mouse Serum Albumin , 1:1000 abcam 34807

Validation

All commercially available antibodies were validated by vendors. Validation statements are provided on the manufacture's website. Antibodies used in the flow cytometry and immunohistochemistry analysis studies have been further validated in the following publications from our laboratory.
1) https://www.ncbi.nlm.nih.gov/pubmed/28129117
2) https://www.ncbi.nlm.nih.gov/pubmed/30721028

Eukaryotic cell lines

Policy information about cell lines

Cell line source(s)	Mouse (GL26, GL26-OVA, GL26-cit)
Authentication	The cell lines used in the study were no authenticated.
Mycoplasma contamination	All cell lines used in the study were mycoplasma contamination free.
Commonly misidentified lines (See ICLAC register)	We did not use any commonly misidentified cell lines in the study.

Animals and other organisms

Policy information about studies involving animals; ARRIVE guidelines recommended for reporting animal research

Laboratory animals	Six to eight week old female C57BL/6 mice were purchased from Jackson Laboratory (Bar Harbor, ME) and were housed in pathogen free conditions at the University of Michigan.
Wild animals	No wild animals were used.
Field-collected samples	No field-collected samples were used.

Ethics oversight	All experimental studies were performed in compliance with the University of Michigan Institutional Animal Care & Use Committee (IACUC).

Note that full information on the approval of the study protocol must also be provided in the manuscript.

Flow Cytometry

Plots

Confirm that:

- [x] The axis labels state the marker and fluorochrome used (e.g. CD4-FITC).
- [x] The axis scales are clearly visible. Include numbers along axes only for bottom left plot of group (a 'group' is an analysis of identical markers).
- [x] All plots are contour plots with outliers or pseudocolor plots.
- [x] A numerical value for number of cells or percentage (with statistics) is provided.

Methodology

Sample preparation	Mice were euthanized and the tumor mass within the brain was dissected and homogenized using Tenbroeck (Corning) homogenizer in DMEM media containing 10% FBS. Tumor infiltrating immune cells was enriched with 30%-70% Percoll (GE Lifesciences) density gradient and the cells were resuspended in PBS containing 2% FBS (flow buffer). Live/dead staining was carried out using fixable viability dye (eBioscience). Non-specific antibody binding was blocked with CD16/CD32. Dendritic cells were labeled with CD45, CDllc, and B220 antibodies. Plasmacytoid dendritic cells (pDCs) were identified as CD45+/CD11c +/ B220+ and conventional dendritic (cDCs) cells were identified as CD45+/CD11c+/B220-. Macrophages were labeled with CD45, F4/80, and CD206 antibodies. Ml macrophages were identified as CD45+/ F4/80+/ CD2061ow and M2 macrophages were identified as CD45+/ F4/80+/ CD206high. Tumor specific T cells were labeled with CD45, CD3, CD8 and SIINFEKL-H2Kb-tetramer. Granzyme B and IF Ny were stained using BD intracellular staining kit following the manufacturer's instructions. For T cell functional analysis, purified immune cells from the tumor microenvironment were stimulated with 100 µg/ml of GL26-OVA lysate for 16 hrs in DMEM media containing 10% FBS followed by 6 hrs incubation with Brefeldin and monensin. For integrin avS3 and avS5 analysis, untreated GL26-tumor bearing mice were euthanized 23 dpi and both tumor bearing hemisphere and the contralateral hemisphere were dissected. Cells were dissociated from both the hemispheres into single cell suspension and CD45 cells were labeled with magnetic beads (Miltenyi) using the manufactures' instructions at 4 °C. Purified cells were washed and passed through a preconditioned MS column placed in the magnetic field of a MACS separator. Cells that were negative for CD45 were collected, resuspeneded in flow buffer and labeled with avS3 (Novus, NBP2-67557) and avS5 (BD Bioscience, 565836) for flowcytometry analysis. All stains were carried out for 30min at 4°C with 3X flow buffer washes between live/dead staining, blocking, surface staining, cell fixation, intracellular staining and data measurement.
Instrument	BD FACSAria'" Ill
Software	Flow Jo version 10 (Treestar)
Cell population abundance	Cells were not sorted for experiments detailed in the manuscript. For each staining carried in this study, 10,000-30,000 events were recorded for viable cells. Live/dead staining was carried out using fixable viability dye (eBioscience).
Gating strategy	The following gating strategy was used for experiments detailed in the manuscript: FSC-A vs. SSC-A➔ SSC-H vs. SSC-W ➔ FSC-H vs. FSC-W ➔ CD45+/- vs. Live/Dead ➔ Stain of interest. Plasmacytoid dendritic cells (pDCs) were identified as CD45+/ CD11c+/ B220+ and conventional dendritic (cDCs) cells were identified as CD45+/CD1lc+/B220-; M 1 macrophages were identified as CD45+/ F4/80+/ CD2061ow and M2 macrophages were identified as CD45+/ F4/80+/ CD206high; Tumor specific T cells were identified as CD45+/CD3+/CD8+/SIINFEKL-H2Kb-tetramer+; Activated T cells were identified as CD45+/CD3+/CD8 +/IFNy+. Activated dendritic cells in the draining lymph node were identified as CD45+/CD11c+/MHC II+ . Positive and negative cells for each stain were defined by fluorescence minuses one control method.

- [x] Tick this box to confirm that a figure exemplifying the gating strategy is provided in the Supplementary Information.
